# Altered Dynamic Postural Stability and Joint Position Sense Following British Army Foot-Drill

**DOI:** 10.3389/fspor.2020.584275

**Published:** 2020-10-22

**Authors:** Alex J. Rawcliffe, Katrina L. Hinde, Scott M. Graham, Russell Martindale, Andrew Morrison, Kellen T. Krajewski, Chris Connaboy

**Affiliations:** ^1^Head Quarters Army Recruiting and Initial Training Command, Ministry of Defence, London, United Kingdom; ^2^Defence Science and Technology Laboratory, Porton Down, Salisbury, United Kingdom; ^3^School of Applied Sciences, Edinburgh Napier University, Edinburgh, United Kingdom; ^4^Cambridge Centre for Sport and Exercise Sciences, Anglia Ruskin University, Cambridge, United Kingdom; ^5^Neuromuscular Research Laboratory, University of Pittsburgh, Pittsburgh, PA, United States

**Keywords:** injury risk, female recruits, neuromuscular function, balance, occupational military activity

## Abstract

Impaired proprioceptive acuity negatively affects both joint position sense and postural control and is a risk factor for lower-extremity musculoskeletal injury in athletes and military personnel. British Army foot-drill is an occupational military activity involving cyclical high impact loading forces greater than those observed in athletes during high level plyometrics. Foot-drill may contribute to the high rates of lower-extremity overuse injuries observed in recruits during basic training. There is limited research investigating foot-drill specific injury risk factors in women, despite greater incidences of musculoskeletal injury reported in women (522 vs. 417 per 1,000 personnel, OR: 1.53) when compared to men during basic training. This study aimed to quantify changes in ankle joint proprioception and dynamic postural stability following a period of British Army foot-drill. Fourteen women of similar age to British Army female recruits underwent pre-post foot-drill measures of frontal plane ankle joint position sense (JPS) and dynamic postural stability using the dynamic postural stability index (DPSI). Passive ankle JPS was assessed from relative test angles of inversion 30% (IN30%) and eversion 30% (EV30%) and IN60% of participants range of motion using an isokinetic dynamometer. The DPSI and the individual stability indices (medio-lateral [MLSI], anterior-posterior [APSI], and vertical [VSI]) were calculated from lateral and forward jump-landing conditions using force plates. Foot-drill was conducted by a serving British Army drill instructor. Significantly greater absolute mean JPS error for IN30% and EV30% was observed post foot-drill (*p* ≤ 0.016, *d* ≥ 0.70). For both the lateral and forward jump-landing conditions, significantly greater stability index scores were observed for MLSI, APSI, and DPSI (*p* ≤ 0.017, *d* ≥ 0.52). Significantly greater JPS error and stability index scores are associated with the demands of British Army foot-drill. These results provide evidence that foot-drill negatively affects lower-extremity proprioceptive acuity in recruit age-matched women, which has implications for increased injury risk during subsequent military physical activity, occurring in a normal training cycle.

## Introduction

The aim of initial military training or Phase one Basic Training (BT) is to transform civilians into trained soldiers. The British Army provides several intense physical training programmes that prepares recruits for combat. Part of the BT syllabus involves recruits performing many hours of British Army foot-drill, or foot-drill training; a fundamental occupational military activity that is frequently practiced by recruits during BT (Rawcliffe et al., 2020). Foot-drill has been suggested as a potential contributing risk factor for lower-extremity musculoskeletal injury. British Army foot-drills are characterized by their own unique movement patterns; quick-march involves marching at two paces per second whilst impacting the ground with an exaggerated heel strike; stand-at-attention, stand-at-ease, halt and about-turn (left and right) all involve raising the active limb to 90-degree (°) hip flexion and forcefully stamping down onto the ground with an extended-knee (i.e., straight-leg landing). It is these regimental movement patterns that have been implicated in the high impact loading forces and tibial accelerations of foot-drill irrespective of sex, experience (i.e., trained [soldiers] vs. untrained [recruits]) (Carden et al., 2015) and footwear (drill shoe vs. combat boot and gym training shoe) (Rawcliffe et al., 2020).

Carden et al. (2015) investigated the force and acceleration characteristics of foot-drill in trained (i.e., soldiers) and untrained (i.e., recruits) men and women, reporting peak vertical ground reaction forces (vGRF) [1.3–6.6 bodyweights (BW)], loading rates (42–983 BW/sec), and peak tibial impact accelerations (23–207 m/s^2^). Rawcliffe et al. (2020), Rawcliffe et al. (2016), and Connaboy et al. (2011) all reported similar magnitudes of impact loading forces for recruit age-matched civilian men and women. However, these studies used observational lab-based study designs and assessed foot-drills independent of each other, therefore lacking ecological validity of the cumulative impact loading forces of foot-drill. To date, only one study has assessed cumulative lower-extremity loading of foot-drill in real-time during BT. Rice et al. (2018) used shank-mounted (tri-axial) tibial accelerometers to quantify estimates of lower-extremity loading in the field. Repetitive impacts at high [>10 gravitational accelerations (g)] and very high (>15 g) tibial shock magnitudes were observed for both male and female recruits, with peak positive accelerations and mean peak positive accelerations exceeding the g threshold of the device (±16 g). Despite known limitations of extrapolation (i.e., accuracy), these values repeatedly exceeded 16 g and are greater than values reported during running (LaFortune, 1991) and plyometric exercises (i.e., single-leg drop landings) (Coventry et al., 2006); the latter being a training modality more commonly associated with more experienced and better conditioned athletes (Connaboy et al., 2011) due to the high risk of MSK injury associated with this type of activity (Davies et al., 2015).

Altered and/or diminished joint proprioception and postural stability, as measured by joint positional sense (JPS) and the dynamic postural stability index (DPSI), have been prospectively identified as risk factors for lower-extremity injury in athletic and recreational active populations (McGuine et al., 2000; Trojian and McKeag, 2006; Mckeon and Hertel, 2008; Ross et al., 2009; Sell et al., 2013; LaGoy et al., 2020) and are likely key risk factors for injury in military recruits during BT. Prospective studies have reported significant reductions in joint proprioceptive acuity and postural stability following military specific exercise (Mohammadi et al., 2013; Sell et al., 2013) and during high impact activity (i.e., plyometrics) similar to that of foot-drill (Twist et al., 2008). Indeed, latent impairments in lower-extremity neuromuscular function following high impact activity have been reported (Twist et al., 2008). However, it is unknown whether the high impact loading forces and regimented movement patterns of British Army foot-drill attenuate the acuity of lower-extremity neuromuscular control, which may have implications for the use of skill-based activities (i.e., obstacle course) and increased injury risk during subsequent BT activities.

Research investigating military training-related injury risk factors specific to female recruits is limited, despite female recruits demonstrating a two-to-three times greater risk of lower-extremity musculoskeletal injury during BT when compared to their male counterparts (Strowbridge and Burgess, 2002; Blacker et al., 2008; Wilkinson et al., 2011; Ministry of Defence, 2016). This is corroborated in the athletic literature, where athletic females demonstrate a four-to-six times greater incidence of anterior cruciate ligament injury (Arendt et al., 1999) and lateral ankle sprains (Hosea et al., 2000) while participating in the same sporting activities as men. Lower-limb sex differences demonstrate that exercising females are generally ligament dominant (i.e., the absence of muscle control of medio-lateral joint motion resulting in greater joint torques and vGRF) (Hewett et al., 2002), employ different landing strategies (Wikstrom et al., 2006), and demonstrate neuromuscular imbalances between dominant and non-dominant lower-limbs (Decker et al., 2003). These predisposing injury risk factors may place female recruits at greater risk of impaired neuromuscular control as measured by JPS and the DPSI following British Army foot-drill training.

The aim of this study was to quantify changes in ankle JPS acuity and DPSI [including stability indices (medio-lateral and anterior-posterior)] pre and immediately post a period of British Army foot-drill in women of similar age to female recruits. It was hypothesized that women would experience significantly greater absolute JPS error of the ankle joint and increased dynamic postural variability from DPSI (and stability indices) post foot-drill.

## Methods

### Participants

Participants aged between 18 and 32 years (as per British Army basic adult solider entry age requirements) were considered for this study. Fourteen women (university students) of similar age to British Army female recruits (*n* = 14, age: 26 ± 3 yrs, height: 179.2 ± 6.2 cm, body mass: 74.4 ± 2.6 kg) were successfully recruited for this study. All participants were recreationally active, taking part in moderate physical activity or sport a minimum of two-to-three times per week, defined as “untrained” as participants obtained no prior experience of British Army foot-drill. Participants reported no injuries or pathological lower-limb, hip or spinal conditions prior to testing, no prior history of balance, jump-landing or foot-drill training, no neurological or vascular compromise, and no known pregnancy at the time of testing. All but two participants of this study were right foot dominant. Ethical approval was gained from Edinburgh Napier University's local ethics committee.

## Experimental Design

This observational study quantified changes in ankle joint proprioception and dynamic postural stability pre and post a period of British Army foot-drill training. To mitigate potential learning effects, participants performed a single familiarization session involving multiple practice trials of ankle JPS and dynamic postural stability (Hopkins, 2000) whilst wearing standard issue British Amy footwear (combat boots) the day before data collection. Ankle JPS and dynamic postural stability data were collected and analyzed from the dominant limb only, defined as the limb used to strike a ball. Measures of ankle JPS were conducted prior to DPSI as to mitigate the effects of jump-landing activity on measures of ankle JPS.

### British Army Foot-Drill Training

A serving British Army foot-drill instructor conducted each standardized foot-drill session, relative to the British Army foot-drill instructor manual. Each session lasted ~88 min (Table 1) with JPS and DPSI conducted pre and immediately post foot-drill training. Foot-drills are characterized by their own unique key performance markers (The Rifles Drill Manual, 2017). For example, quick-march involves marching at two paces per second whilst impacting the ground with an exaggerated heel strike. The stand-at-attention, stand-at-ease, right-turn, about-turn (left-leg), left-turn, and halt foot-drill (right-leg) involves raising the active limb ~90° hip flexion and forcefully stamping onto the ground, with an extended-knee (straight-leg) landing. During each foot-drill session, participants wore standard issue British Army footwear provided by the research team.

**Table 1:** Frequency (repetitions), duration (time), and the total *n* of impacts performed with the right and left leg during the standardized period of foot-drill.

**Foot-drill**	**British Army foot-drill**		
	**Duration (mins)**	***n* left foot impacts**	***n* right foot impacts**
Stand-at-attention	11	42	-
Stand-at-ease	9	28	-
Right-turn	12	48	-
Left-turn	9	-	32
About-turn	10	26	-
Halt	18	-	39
March	12	128	118
Rest	7	-	-
Total	88	272	189

### Ankle Joint Position Sense (Passive)

Frontal plane [Inversion/Eversion (IN/EV)] ankle JPS was quantified using a Biodex dynamometer (Biodex Medical Systems, Shirley, New York, USA) using methods described previously (Brown et al., 2004; Sefton et al., 2009). Ankle JPS was assessed in the frontal plane rather than the sagittal plane as most injuries occur around the anterior-posterior axis (i.e., lateral ankle sprain). The test ankle was positioned in a clinically designated neutral or 0° position, achieving 90° between the foot and tibia. Participants were blindfolded and wore headphones to eliminate any contribution of visual and audio cues to the positioning of the test ankle. Participants were given a 45 second (sec) recovery between trials to mitigate fatigue and to assist with concentration. Ankle IN/EV range of motion (ROM) was determined prior to testing. From which, 30 and 60% of full inversion ROM and 30% of full eversion ROM of each participant was calculated and utilized as JPS test angles. This accounted for relative ankle joint flexibility whilst reducing the effect of additional sensory input from cutaneous receptors at extreme ROM (Burke et al., 1988). At random, the test ankle was passively moved into one of three test positions, 30 and 60% IN and 30% EV. Each test angle was locked in position for 10 s and passively moved through its respective ROM (60°/s) before returning to neutral (0°). Participants were required to match the previously presented test angle and press a handheld trigger that recorded the absolute degrees of error (°) between the test angle and reproduced angle. The mean of three trials from each IN/EV JPS condition pre-post foot-drill training was collected and processed for further analysis.

### Dynamic Postural Stability

Similar to methods used previously (Sell et al., 2013), ground reaction force (GRF) data was collected at 1,000 Hz *via* a Kistler force plate (Kistler Instruments AG, 9281CA, Switzerland). The DPSI has been found to have a high intra-session reliability and to be a precise measure (ICC−0.86, SEM = 0.01) (Sell et al., 2013). Dynamic postural stability was assessed from an anterior-posterior (A/P) and medio-lateral (M/L) jump-landing task and analyzed using the DPSI. Relative to the A/P and M/L jump-landings, female participants stood bilaterally at a distance of 40 and 33% of their standing height from the middle of the force plate, respectively. When instructed, participants jumped anteriorly (A/P jump) or laterally (M/L jump) off both legs, over a 12 inch (A/P jump) or 6 inch (M/L jump) hurdle, landing on the force plate with the dominant-limb (single-leg landing). Participants were asked to stabilize immediately after landing, placing both hands on hips and balancing for 13 s (Wikstrom et al., 2005). Upper-limb movement was not restricted during the take-off or flight phase of each task. Dynamic trials were discarded and repeated if the participants' non-stance limb contacted the stance limb or the ground surrounding the force plate. The mean of three trials from each jump-landing condition (A/P and M/L) pre-post foot-drill training was collected. Ground reaction force data was extracted from the force plate using Bioware® (5.3.0.7 systems) for subsequent analysis.

## Data Analysis

### Dynamic Postural Stability Index

All dynamic postural stability data were treated using a 4th order (zero-lag) low pass Butterworth filter with a cut-off frequency of 20 Hz (Williams et al., 2016). The DPSI and its directional components [stability index: medial lateral (MLSI), anterior-posterior (APSI), vertical (VSI)] were analyzed using a custom Matlab script file. These indices are mean square deviations assessing fluctuations around a 0 point, rather than SDs assessing fluctuations around a group mean (Sell et al., 2013). The MLSI and APSI directional components analyse the fluctuations from zero along the X (A/P) and Y (M/L) axis. The VSI assesses the fluctuations from the participant's bodyweight (as a zero point) along the Z (vertical) axis of the force plate (**Equations 1–3**). The DPSI is a composite of the MLSI, APSI, and VSI, therefore is sensitive to changes in each directional component and is a unitless measure. The DPSI was determined from using the first 3 s of the GRF immediately following initial contact, identified as the instant the vGRF exceeded 5% BW. Greater stability index (SI) scores reflect greater variability and potentially altered dynamic postural stability, with MLSI, APSI, VSI, and DPSI calculated as (BW in newtons[N]);

(1) $$\begin{array}{l} \text{MLSI}=\sqrt{\left(\frac{\sum {\left(0-GRFx\right)}^{2}}{number\;of\;data\;points}\right)}÷BW \end{array}$$

(2) $$\begin{array}{l} \text{APSI}=\sqrt{\left(\frac{\sum {\left(0-GRFy\right)}^{2}}{number\;of\;data\;points}\right)}÷BW \end{array}$$

(3) $$\begin{array}{l} \text{VSI}=\sqrt{\left(\frac{\sum {\left(BW-GRFz\right)}^{2}}{number\;of\;data\;points}\right)}÷BW \end{array}$$

(4) $$\begin{array}{l} \text{DPSI}=\sqrt{\left(\frac{\sum {\left(0-GRFx\right)}^{2}+\sum {\left(0-GRFy\right)}^{2}+\sum {\left(0-GRFz\right)}^{2}}{number\;of\;data\;points}\right)}÷\;BW \end{array}$$

### Statistical Analysis

**Figure 1:**
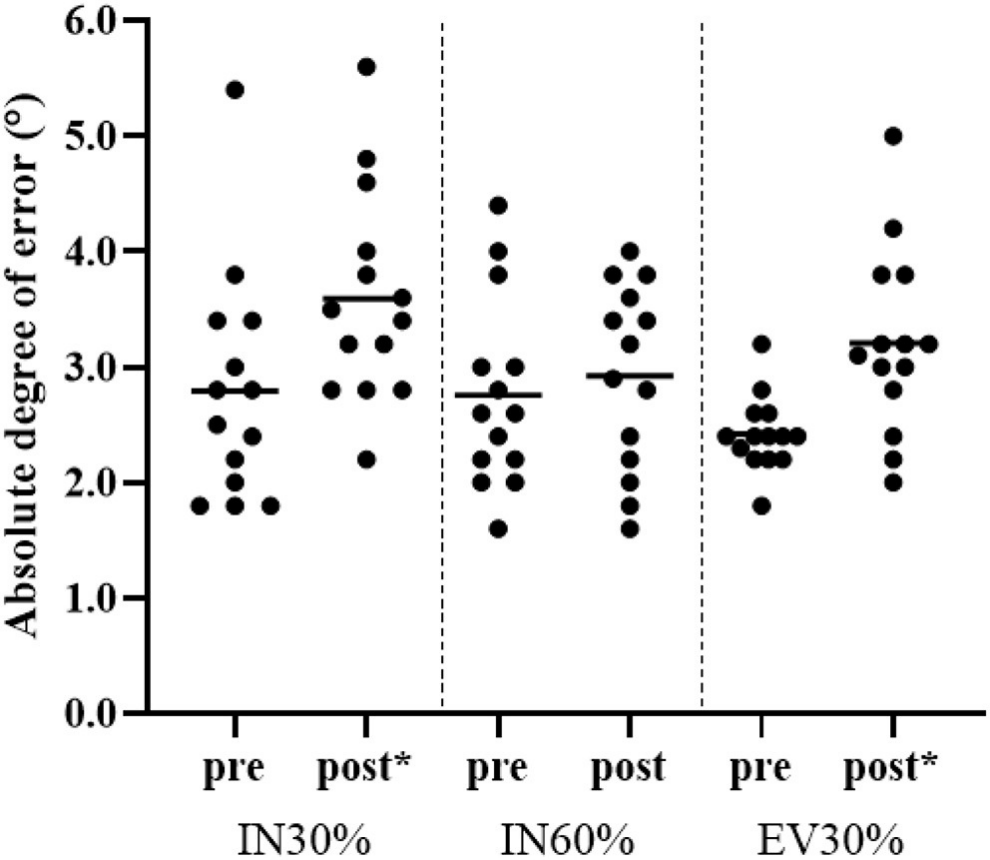
Absolute degree of error (°) for IN30%, IN60%, and EV30% pre—post foot-drill. Mean data is shown by a solid horizontal line. *denotes a significant increase in JPS score post foot-drill.

Mohammadi et al. (2013) demonstrated significantly greater absolute ankle JPS error following military specific exercise reporting large effects (Δ% = 29%, *d* ≥ 1.0). Similarly, LaGoy et al. (2020) reported significantly greater dynamic postural stability index scores during military specific exercise (Δ% = 10%, *d* = 0.83) reporting large effects. Based on these findings, an *a priori* G^*^Power (v3.1.9.2, Germany) calculation was used to estimate sample size for two-sided paired-samples t-test to statistically determine differences in JPS and dynamic postural stability pre - post a period of British Army foot-drill. With an estimated 20% probability (power = 0.80) of a type II error, an alpha level set at 0.05, and an estimated effect size of 0.83 (LaGoy et al., 2020), it was estimated that a sample size of 14 female participants would be sufficient to detect a significant difference from zero. Mean ± *SD* for each dependant variable (DV) were calculated (Figure 1 and Table 2). Each DV was examined for normality. Data were analyzed from the dominant limb only and averaged across three-trials for each JPS test angle and dynamic postural stability jump-landing condition. A series of paired samples t-tests were conducted to determine differences in JPS data and differences in dynamic postural stability (pre vs. post foot-drill). Cohens d effects sizes were also calculated using the following criteria (0.2 = small, 0.5 = medium, 0.8 = large, >0.8 = very large) (Cohen, 1988). Statistical significance was accepted as *p* ≤ 0.05.

**Table 2:** Normalized mean ± *SD* for DPSI and stability indices pre—post foot-drill for M/L and A/P jump-landing conditions (A/P, anterior-posterior; M/L, medio-lateral).

**Jump condition**		**Stability index**			
		**MLSI**	**APSI**	**VSI**	**DPSI**
M/L Jump	Pre	0.019 ± 0.002	0.008 ± 0.003	0.289 ± 0.033	0.284 ± 0.032
	Post	0.097 ± 0.008^a^	0.035 ± 0.007^a^	0.290 ± 0.041	0.308 ± 0.039^a^
	%Δ (*pre-post)*	*433.0 ± 88.2*	*410.4 ± 147.6*	*0.4 ± 8.4*	*6.6 ± 8.1*
A/P Jump	Pre	0.006 ± 0.001	0.023 ± 0.002	0.304 ± 0.037	0.305 ± 0.037
	Post	0.028 ± 0.005^a^	0.121 ± 0.013^a^	0.305 ± 0.033	0.329 ± 0.033^a^
	%Δ (*pre-post*)	*382.2 ± 100.8*	*422.0 ± 67.0*	*0.8 ± 9.0*	*8.7 ± 9.0*

a *Denotes a significant difference from pre-values (p < 0.05); mean* ± *SD percentage change (%*Δ*)*.

## Results

### Joint Positional Sense (JPS)

Figure 1 shows the mean absolute JPS error for each test angle pre—post foot-drill. Significant increases in IN30% (mean difference = 0.80°, *p* < 0.01, *d* = 0.84, 95%CI: 0.04, 1.58) and EV30% (mean difference = 0.79°, *p* = 0.009, *d* = 1.72, 95%CI: 0.81, 2.54) were observed post foot-drill. There was no significant change in IN60% values (mean difference = 0.16°, *p* = 0.618, *d* = 0.24, 95%CI: 0.51, 0.98).

### Dynamic Postural Stability Index (DPSI)

Table 2 shows the A/P jump-landing condition, MLSI (*p* < 0.001, *d* = 6.45, 95%CI: 4.04, 7.74), APSI (*p* < 0.001, *d* = 10.46, 95%CI: 7.19, 13.16), and DPSI (*p* = 0.006, *d* = 0.70, 95%CI: 0.16, 1.48) were significantly greater post foot-drill. Similarly, MLSI (*p* < 0.001, *d* = 13.38, 95%CI: 9.18, 16.66), APSI (*p* < 0.001, *d* = 5.38, 95%CI: 3.25, 6.43), and DPSI (*p* = 0.017, *d* = 0.52, 95%CI: 0.17, 1.47) were significantly greater post foot-drill for the M/L jump landing condition. There were no significant changes in VSI for the A/P (*p* = 0.906, *d* = 0.03, 95%CI: −0.83, 0.77) or M/L jump-landing conditions (*p* = 0.871, *d* = 0.03, 95%CI: −0.83, 0.77).

## Discussion

This is the first study to examine potential deficits in lower-extremity neuromuscular function following a period of British Army foot-drill. In agreement with our hypothesis, significantly greater absolute JPS error was observed for IN30% and EV30% post foot-drill, demonstrating a large effect of foot-drill on smaller (*d* ≥ 0.84) versus larger (*d* = 0.24) JPS test angles. Participants demonstrated a 29 and 32% increase in absolute JPS error post foot-drill for IN30% and EV30%, respectively. Although an increase in absolute JPS error for IN60% (6%) was observed, no significant differences were reported, and the size of the effect was considered trivial. Significantly greater GRF variability following foot-drill in DPSI, MLSI, and APSI for both the M/L and A/P jump-landing conditions was observed. The magnitude of differences (%) in pre-post foot-drill measures of dynamic postural stability were very high (see Table 2), with effect sizes ranging from medium to very large (*d* = 0.52 – 13.38). The differences in the composite DPSI (an overall score reflective of changes in directional components) are likely from changes in the APSI and MLSI, as no significant differences were observed for VSI post foot-drill for either of the jump-landing conditions.

Ankle injury is among the most common MSK injuries reported in athletes and Army recruits during routine training conditions (Andersen et al., 2016). Joint position sense is commonly used as a functional measure of proprioception as it plays a key role in maintaining dynamic stability of lower-extremity joints and has been shown to predict ankle injury in uninjured male and female athletic populations (Payne et al., 1997; Willems et al., 2005b). Acute trauma is a key factor in some injury cases, resulting in high rates of recurrence and frequently leading to disruption of ligamentous joint afferents and loss of proprioceptive acuity (Willems et al., 2002; Clark et al., 2015; Röijezon et al., 2015). However, many lower-extremity injuries reported in BT result from the cumulative effects of microtraumatic forces associated with overtraining, repetitive and high impact movements, extreme joint positions and prolonged static positioning (Hauret et al., 2010; Mohammadi et al., 2013). This is common for British Army foot-drill, involving long and frequent periods of static upright positioning and impacting the ground repeatedly with extreme joint positions (i.e., extended-knee landings while intentionally mitigating hip and knee flexion at impact).

Studies investigating changes in lower-extremity neuromuscular function relative to military specific exercises are limited. However, to the authors knowledge, only one other study has investigated changes in absolute JPS error following military specific exercise. Mohammadi et al. (2013) reported significantly greater absolute JPS error of the ankle joint (using similar methods) in military conscripts immediately following military specific exercise. In our study, participants demonstrated a 0.80° increase in absolute JPS error for both IN30% and EV30% following foot-drill, with large effect sizes. Similarly, Mohammadi et al. (2013) reported significant differences and large effect sizes for increases in absolute JPS error of 0.70° immediately post military specific exercise. It was further (descriptively) reported that conscripts who sustained an injury after 8- weeks of BT (hamstring and ankle sprains, ACL rupture and stress fracture of the metatarsals) demonstrated significantly greater absolute JPS error (mean Δ = 2°) compared to uninjured conscripts. Indeed, deficits in proprioception are shown to be predictive of injury in uninjured, physically active populations (Payne et al., 1997). However, due to insufficient study power (i.e., small sample, *n* = 8) reported by Mohammadi et al. (2013), it is unknown whether an increase in absolute JPS error is predictive of ankle MSK injury in military recruits during BT. Additionally, the specific type of military exercises that led to reductions in ankle JPS acuity were not reported, and in turn, limits our understanding of the potential effects of common military specific exercises on injury risk. We must consider that although a significant increase in JPS error was observed post foot-drill, this increase was <1° and the clinical implications of this small increase in absolute JPS error remain unclear.

Comparable to our study, South and George (2007) reported no significant mean differences in absolute JPS error for larger (90% of ROM) IN test angles pre-post fatiguing activity. A possible explanation for the smaller absolute JPS error (0.16°) observed for IN60% post foot-drill may be due to greater joint torque found with greater test angle positions. Studies show that as joint torque demand increases, there is a high potential to increase proprioceptive acuity (Bullock-Saxton et al., 2001; Suprak et al., 2007; Lyons and Lyons, 2017). In our study, it is possible that the added weight from the foot-plate (and gravity) combined with the greater test angle of IN60% produced a greater theoretical moment arm, resulting in greater joint torque and tension of surrounding muscles. With increased joint torque, an increase in muscle activation (specifically alpha and gamma motor neurons) is observed, thereby increasing the sensitivity of intramuscular receptors (i.e., GTO) that relay proprioceptive feedback during movement. Test angles near to maximum ROM (i.e., 90%IN/EV) are defined as extreme test angles and are considered a limitation due to the effect of additional sensory input from cutaneous receptors on the ability to reproduce the test angle (Burke et al., 1988). The average IN/EV ROM has been identified as 30 and 18°, respectively (Ball and Johnson, 1996). In our study, we employed test angles of IN60% and IN/EV30% of each participants ROM, which corresponds to an ~18° IN and 9° EV test angle based on the average IN/EV ROM. Although IN60% is not considered an extreme test angle, this test angle lies much closer to the ankles average end ROM than 30%. Therefore, the reduced absolute JPS error for IN60% observed in our study is likely associated with increased muscle activation from greater joint torque demand at this position. Furthermore, these data suggest, in part, that as inversion angles approach their end ROM, an individual's JPS acuity will improve (i.e., reduced absolute JPS error). It is acknowledged, however, that this study was unable to collect data to determine the precise mechanisms associated with the greater and lower absolute JPS error for IN/EV 30% and IN60% respectively, post foot-drill.

To date, no study has quantified changes in measures of dynamic postural stability post military specific exercise. However, changes in dynamic postural stability have been reported for military related tasks. Sell et al. (2013) reported significantly greater stability index scores with the addition of body armor. Increases were identified in all stability indices, including VSI, indicating that with the addition of tactical body armor (~12 kg) greater GRF variability is observed, inferring diminished dynamic postural stability. In our study, no significant changes in the VSI were found, only significantly greater stability index scores (reflecting greater GRFs) were observed for MLSI, APSI and DPSI for both the M/L and A/P jump landing conditions. The greater stability index scores observed post foot-drill for MLSI and APSI during the M/L and A/P jump landing condition respectively, may have placed participants closer to their limits of stability, reflecting greater displacement of the center of mass and necessitating greater frontal and sagittal plane control (Meardon et al., 2016). As mentioned earlier, differences in the composite DPSI appear to largely reflect changes in the APSI and MLSI as no differences in VSI were observed post foot-drill. The significantly greater VSI reported by Sell et al. (2013) is likely related to the additional load from the body armor. However, it is possible that changes in dynamic postural stability reported in our study may be due, in part, to the effects of fatigue resulting in potential changes in muscle activation patterns and lower-extremity jump-landing kinematics (Wikstrom et al., 2005; Sell et al., 2013; Meardon et al., 2016). Indeed, the effects of fatigue on lower-extremity kinematics during jump-landing activities has been well-reported in athletic females (Benjaminse et al., 2007; Luccia et al., 2011; Cortes et al., 2013; Lessi et al., 2017). Since lower-extremity kinematic and EMG data were not collected during our study, we cannot confirm whether increased dynamic postural stability index scores (inferring impaired stability) observed post foot-drill was related to the effects of fatigue on landing kinematics and muscle activation patterns. Therefore, further research is warranted to elucidate these claims.

A greater dynamic postural stability index infers increased GRF variability during stabilization following a landing task (Wikstrom et al., 2005). Greater stability indices are typically considered as an indicator of poorer postural stability and impaired neuromuscular function (Sell et al., 2013). This presumption is supported by others reporting increased variability with increased balance task demand (Goldie et al., 1989). Additionally, increased dynamic postural stability has been identified as a risk factor for lower-extremity MSK injury and shown to predict injury in uninjured athletic populations (McGuine et al., 2000; Willems et al., 2005a; Trojian and McKeag, 2006; Wang et al., 2006). Recently, traditional perspectives of increased variability within biological systems has been challenged based on non-linear dynamics, commonly referred to as the chaos theory, which associates high variability with a more functional and adaptable system (van Emmerik and van Wegen, 2002; Meardon et al., 2016). Therefore, it is recommended that the interpretation of these variability measures be considered in conjunction with other validated measures of neuromuscular function.

A number of limitations of this study are acknowledged. In our study, we did not collect data from British Army recruits on repeated measures of JPS and dynamic postural stability to quantify the transient effects of foot-drill on neuromuscular function. However, based on the literature, it is possible that military recruits may experience prolonged impairments in neuromuscular function as a consequence of foot-drill training (Yaggie and McGregor, 2002; Paschalis et al., 2007). As such, further research is warranted to determine the extent of change in joint movement and position in recruits specifically, as these results may have important implications for subsequent skill-based military activities (i.e., obstacle course), scheduling of high intense training and recovery sessions, and injury risk. Although foot-drill is considered an injury risk factor in both men and women, our study did not compare pre-post foot-drill measures of JPS and dynamic postural stability between sex. However, women generally demonstrate greater risk and incidence of injury compared to their male counterparts (Wikstrom et al., 2006), and research investigating female specific injury risk factors associated with the demands of foot-drill and other occupational military activities are limited, despite the growing role of women in the Armed Forces. It is acknowledged, however, that understanding the risk of injury associated with the demands of occupational military activities (such as foot-drill) using robust methodology is challenging to implement in a military setting, due to the additional burden and disruption to military training programmes, while controlling for many other confounding factors that are likely to contribute to the risk of injury during BT.

Impaired neuromuscular function has been shown to alter lower-extremity kinematics associated with injury risk (Benjaminse et al., 2007; Luccia et al., 2011; Cortes et al., 2013; Lessi et al., 2017). Unfortunately, we did not collect data on lower-extremity kinematics and muscle activation patterns, nor did we determine the level of fatigue (both cognitive and physiological) of participants post foot-drill. In our study, the effects of muscle fatigue have been implicated in the greater absolute JPS error and dynamic postural stability observed post foot-drill. Given that both muscle and cognitive fatigue have been linked with reductions in neuromuscular function and altered lower-extremity biomechanics, further study is warranted to better understand the extent of change in predictors of injury risk following foot-drill with participants in a fatigued state, as losses in neuromuscular function and increases in attentional demand (Bisson et al., 2011) may be exacerbated which has implications for additional risk and increased severity of injury.

## Conclusion

Significantly greater absolute JPS error and greater dynamic postural stability index scores (inferring poorer postural stability) was observed in a cohort of female participants following a period of British Army foot-drill, as evidenced by greater absolute JPS error and increased GRF variability in MLSI, APSI, and DPSI for the M/L and A/P jump-landing conditions. As such, our study suggests that following a period of British Army foot-drill, female recruits may be at an increased risk of lower-extremity injury due to reductions in neuromuscular function observed post foot-drill. These results have implications for the scheduling of subsequent skill-based military activities and recovery sessions to reduce the potential risk of musculoskeletal injury following British Army foot-drill training.

## Data Availability Statement

The raw data supporting the conclusions of this article will be made available by the authors, without undue reservation.

## Ethics Statement

The studies involving human participants were reviewed and approved by Edinburgh Napier University ethics committee. The patients/participants provided their written informed consent to participate in this study.

## Author Contributions

AR: developed study design, collected and analyzed data, and wrote manuscript. KH: assisted with data analysis and development of manuscript. SG and RM: assisted with study design and reviewed manuscript. AM: assisted with data processing/analysis and reviewed manuscript. KK: reviewed manuscript. CC: developed study design in collaboration with AR, provided scripts for data processing and analysis, and reviewed manuscript. All authors contributed to the article and approved the submitted version.

## Conflict of Interest

The authors declare that the research was conducted in the absence of any commercial or financial relationships that could be construed as a potential conflict of interest.
